# Successful application of low-dose regional citrate combined with nafamostat mixed anticoagulation in CRRT for patients with liver failure: a retrospective case series study of 11 patients

**DOI:** 10.3389/fphar.2026.1896125

**Published:** 2026-10-21

**Authors:** Yanting Zhang, Jing Ma, Le Xiao, Jian Ye, Hanqing Li, Zhaoyang Li, Meng Xiao, Xinbo Ding, Pu Zhang

**Affiliations:** 1 Department of Critical Care Medicine, Zhongnan Hospital of Wuhan University, Wuhan, China; 2 Hubei Clinical Research Center for Critical Care Medicine, Wuhan, China

**Keywords:** continuous renal replacement therapy, liver failure, low-dose citrate, mixed anticoagulation, nafamostat, sepsis

## Abstract

**Objective:**

To investigate the clinical efficacy, safety and operational feasibility of a low-dose regional citrate anticoagulation (RCA) combined with low-dose nafamostat mesylate (NM) mixed anticoagulation regimen in continuous renal replacement therapy (CRRT) for patients with liver failure, so as to provide clinical reference for the selection of CRRT anticoagulation strategies for such special high-risk critically ill patients.

**Method:**

A retrospective case series study was conducted. Eleven patients with liver failure who received CRRT in the Department of Critical Care Medicine, Zhongnan Hospital of Wuhan University were enrolled. All patients were treated with CVVHDF mode and uniformly implemented the low-dose citrate combined with nafamostat mixed anticoagulation regimen. CRRT treatment parameters, anticoagulant drugs, and infusion doses of calcium and sodium bicarbonate were standardized. The monitoring frequency of peripheral blood gas and activated partial thromboplastin time (APTT) and the criteria for dose stratification adjustment were standardized. Data including patients' demographic baseline data, CRRT treatment duration, filter service life, blood gas electrolytes, dynamic changes of APTT, pipeline and filter coagulation, bleeding and metabolic adverse events, organ function and clinical outcomes were collected for summary and analysis.

**Results:**

A total of 11 patients with liver failure undergoing CRRT in CVVHDF mode were enrolled in this study, using the low-dose regional citrate combined with nafamostat mixed anticoagulation regimen. After treatment, the median filter life of patients reached 48 (38, 68.75) hours, with no severe pipeline or filter coagulation; there were no significant abnormalities in systemic coagulation indicators. One case (9.1%) had mild oral mucosal bleeding; no severe massive bleeding occurred. Hypocalcemia and metabolic alkalosis were observed in several patients but were not severe, and no citrate accumulation, severe hypocalcemia, or severe acid-base or electrolyte disorders occurred. PCT was lower than baseline (P = 0.005) and APACHE II score was improved (P = 0.011) over the course of ICU treatment; however, all patients simultaneously received antibiotics, vasopressors, source control, and other ICU supportive care, and these changes therefore cannot be attributed to the mixed anticoagulation regimen. Liver and kidney function showed no obvious deterioration.

**Conclusion:**

In this small, single-center retrospective case series, a low-dose regional citrate combined with nafamostat mixed anticoagulation regimen was feasible to implement in patients with liver failure receiving CRRT. In this cohort, the median filter life was 48 h, no severe filter or pipeline coagulation was observed, and no citrate accumulation, severe hypocalcemia, or severe acid-base or electrolyte disorders occurred; one case (9.1%) had mild bleeding. Because no comparator group was included, whether these observations reflect a benefit of the mixed regimen over conventional RCA, nafamostat alone, systemic heparin, or no anticoagulation cannot be determined. These findings are preliminary and hypothesis-generating and require confirmation in adequately powered, multicenter, prospective controlled trials.

## 1 Introduction

Liver failure is a common critical illness in the Department of Critical Care Medicine. Patients have massive hepatocyte necrosis, severe impairment of liver synthesis and metabolic functions, often accompanied by coagulation dysfunction, acid-base and electrolyte disorders of the internal environment, and multiple organ involvement. Continuous renal replacement therapy (CRRT) is often required as early as possible to clear inflammatory mediators, correct internal environment disorders, and replace organ function support therapy (Koch et al., 2017; Chen and Wang, 2023).

The key to the smooth implementation of CRRT is reasonable anticoagulation, and patients with liver failure are a special high-risk group for anticoagulation management. On the one hand, insufficient synthesis of liver coagulation factors and low coagulation function make systemic anticoagulation very likely to cause bleeding (Dhar et al., 2017; Ribic and Crowther, 2016); on the other hand, the liver’s metabolic clearance capacity of citrate is significantly reduced, and conventional-dose regional citrate anticoagulation is prone to citrate accumulation, inducing severe complications such as hypocalcemia and metabolic alkalosis (Kramer et al., 2003; Yu et al., 2020); particularly those with shock, elevated lactate, or more severe liver dysfunction. Single nafamostat is difficult to balance filter anticoagulation and systemic bleeding safety at the same time, and traditional heparin anticoagulation has many complications and poor applicability.

At present, there is no optimal standardized anticoagulation regimen for CRRT in patients with liver failure clinically, and a single anticoagulation method is difficult to meet the three major requirements of effective anticoagulation of filter pipelines, avoiding citrate accumulation, and controlling systemic bleeding risk at the same time. Based on this, this study adopted an innovative low-dose regional citrate combined with nafamostat mixed anticoagulation regimen, and conducted a retrospective analysis of 11 patients with liver failure undergoing CRRT, to explore the clinical efficacy, safety and standardized application process of this regimen, aiming to provide a safe, feasible and replicable clinical protocol for CRRT anticoagulation therapy in critically ill patients with liver failure.

## 2 Methods

### 2.1 Study design and participants

This study was a single-center retrospective case series study. Eleven hospitalized patients diagnosed with liver failure and requiring CRRT in CVVHDF mode admitted to the Department of Critical Care Medicine, Zhongnan Hospital of Wuhan University in 2025 were selected.

Inclusion criteria: Meeting the clinical diagnostic criteria for liver failure; receiving CRRT in CVVHDF mode; complete medical records, CRRT parameters and monitoring follow-up data.

Exclusion criteria: Complicated with active massive hemorrhage requiring emergency hemostatic surgery; allergic to study drugs such as citrate, nafamostat, calcium gluconate; missing key clinical data unable to be included in the analysis.

According to the CMA criteria, liver failure is defined as a severe clinical syndrome characterized by massive hepatocyte necrosis and severe impairment of liver synthesis, metabolism, and detoxification functions. The diagnosis requires the presence of the following: cute onset within 4 weeks (for acute liver failure) or 4–24 weeks (for subacute liver failure), with severe gastrointestinal symptoms such as anorexia, vomiting, and abdominal distension; rogressive jaundice: total bilirubin ≥10× upper limit of normal or daily increase ≥1 mg/dL; Coagulation dysfunction: international normalized ratio (INR) ≥1.5 or prothrombin activity (PTA) ≤40%; epatic encephalopathy of grade II or above (for acute liver failure) or ascites (for subacute liver failure); Exclusion of other causes of liver injury. Or patients with underlying chronic liver disease, the diagnosis of acute-on-chronic liver failure (ACLF) was made when acute hepatic decompensation developed within 4 weeks, meeting the criteria for jaundice, coagulopathy, and complications such as ascites or hepatic encephalopathy, with or without extrahepatic organ failure.

### 2.2 Treatment prescription

All patients were treated with CVVHDF mode, using Fresenius CRRT machines equipped with Ci-Ca module and large-area filters. The low-dose 4% sodium citrate combined with nafamostat mesylate mixed anticoagulation was uniformly adopted (Figure 1). The relevant parameters and management scheme of this CRRT treatment are as follows: blood drawing speed 80 mL/min, treatment blood flow rate 120 mL/min, post-dilution mode adopted, replacement fluid and dialysate flow rate both 1000 mL/h; anticoagulation regimen: continuous infusion of 4% sodium citrate 150 mL/h at the blood drawing end, nafamostat mesylate 20 mg/h before the filter, 5% sodium bicarbonate 100 mL/h before the filter, 10% calcium gluconate at the blood return end, and calcium supplementation speed was set stratification according to peripheral blood calcium level: initial 6 mL/h when blood calcium >1.1 mmol/L, initial 8 mL/h when 1.0–1.1 mmol/L, initial 10 mL/h when 0.9–1.0 mmol/L, initial 12 mL/h when <0.9 mmol/L. In this case, it is necessary to ask the physician for evaluation in time whether additional intravenous calcium supplementation is required. This treatment adopted half-dose regional citrate combined with nafamostat anticoagulation, post-dilution mode to protect the venous drip chamber. Low-dose citrate can reduce the liver metabolic burden. Since the replacement fluid contains calcium, pre-dilution is not used. Routine intervention is not required when post-filter blood calcium is maintained >0.4 mmol/L; anticoagulation goal: peripheral APTT is maintained at 1.2–1.5 times the baseline value to prevent excessive prolongation of indicators and avoid bleeding risk. In terms of monitoring, peripheral blood gas and APTT were detected every 2 h within 6 h after initial startup,and every 4 h after 6 h. Various drug infusion speeds were dynamically adjusted according to the results to maintain the stability of the internal environment; medical staff observed the coagulation status of filters and pipelines and systemic bleeding tendency throughout the process, and simultaneously recorded adverse events such as hypocalcemia, acid-base imbalance, and drug allergy.

**FIGURE 1:**
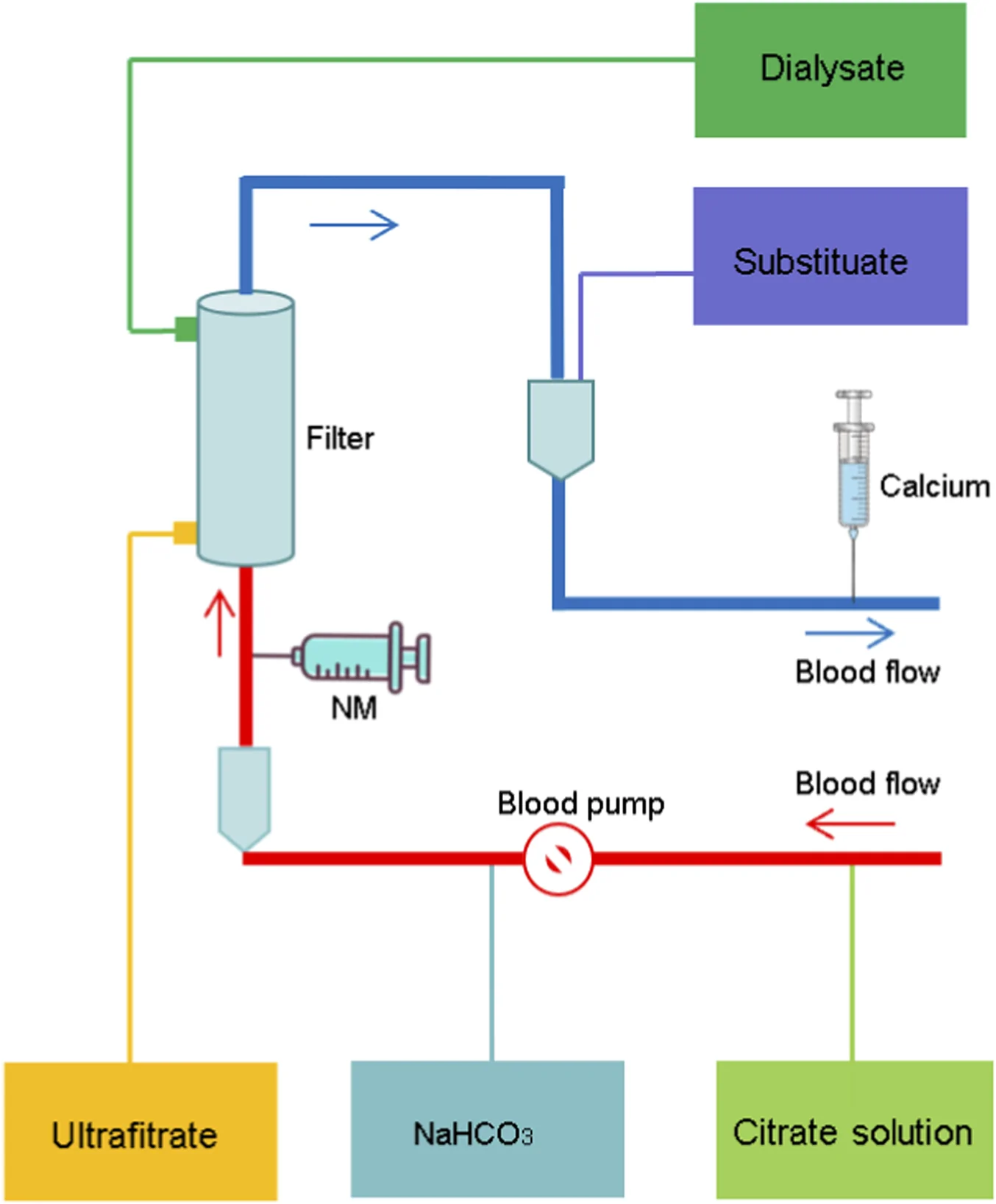
Schematic diagram of CRRT treatment with low-dose 4% sodium citrate combined with nafamostat mesylate mixed anticoagulation (RCA: regional citrate anticoagulation; NM: nafamostat mesylate; NaHCO_3_: sodium bicarbonate).

Sodium bicarbonate (5%) was infused pre-filter at 100 mL/h. This was intended to provide an exogenous buffer source, because citrate metabolism—and therefore endogenous bicarbonate generation—is impaired in patients with liver failure, and to help maintain acid-base stability during low-dose citrate anticoagulation. This bicarbonate dosing strategy was based on institutional experience rather than prospective validation, and its adequacy in this population remains to be evaluated.

### 2.3 Data collection

We report 11 adult ICU patients with liver failure caused by sepsis, tumor, hepatitis, etc., with a median age of 58 (52.25, 66.75) years. All patients were diagnosed with liver failure according to the clinical diagnostic criteria for liver failure (Liver Failure and Artificial Liver Group, Chinese Society of Infectious Diseases, Chinese Medical Association, 2025). Patient characteristics are shown in Table 1. According to the liver failure phenotypes defined by the 2024 CMA criteria, the study comprised 8 patients with ACLF (72.7%; triggered by sepsis/septic shock in 5, cardiogenic-ischemic injury in 2, and malignant tumor in 1), 1 patient with acute liver failure (9.1%; secondary to gastrointestinal massive bleeding), and 2 patients with subacute liver failure (18.2%). Patient-level diagnostic criteria, including phenotype, underlying chronic liver disease, timing of diagnosis, HE grade, INR or PTA, TBil at diagnosis and its trajectory, and other criteria fulfilled, are provided in Supplementary Table S1. All 11 patients fulfilled the formal 2024 CMA diagnostic criteria for liver failure at admission. Figure 2 shows the clinical treatment details of the 11 patients.

**TABLE 1:** Baseline clinical details of patients (n = 11).

Characteristic (baseline)	Patient 1	Patient 2	Patient 3	Patient 4	Patient 5	Patient 6	Patient 7	Patient 8	Patient 9	Patient 10	Patient 11	Total (n = 11) median (IQR), or N (%)
Gender	Female	Female	Male	Male	Male	Male	Female	Male	Male	Male	Female	Female: 4 (36.4%) Male: 7 (63.6%)
Age	51	52	68	62	53	63	50	55	58	80	71	58 (52.25, 66.75)
Cause of liver failure	Sepsis	Gastrointestinal bleeding	Septic shock	Septic shock	Malignant liver tumor	Septic shock	Hepatic encephalopathy	Septic shock	Peritoneal cancer	Cardiogenic shock	Cardiogenic shock	—
BMI, kg/m^2^	16.4	19.29	24.22	24.69	19.84	19.89	22.86	33.3	24.69	25.71	18.73	22.86 (19.43, 24.86)
AKI grade	2	2	2	3	1	3	3	3	3	3	3	Grade 1: 1 (9.1%) Grade 2: 2 (27.3%) Grade 3: 7 (63.6%)
Vasoactive drugs	NE 12 μg/min	None	NE 24 μg/min	NE 160 μg/min	None	NE 4 μg/min	NE 7 μg/min	None	NE 3 μg/min	NE 14 μg/min	None	Yes: 7 (63.6%) No: 4 (36.4%)
ACCI score	7	4	8	9	8	7	6	7	14	7	6	7.17 (6.25, 8.17)
SOFA score	5	7	7	12	6	12	8	5	15	3	4	6.67 (4.83, 10)
APACHE II score	19	12	21	30	20	26	26	14	27	28	26	23.5 (19.25, 26.88)
GCS score	2T	15	2T	2T	15	2T	2T	2T	5T	14	9	4.14 (2, 12.75)
RASS score	1	0	−4	−4	−3	−4	−4	−4	−3	−4	−3	−3.44 (−4, −1.88)
Major surgery (>3 h) within 1 month	No	Yes	No	No	No	Yes	No	No	Yes	No	No	Yes: 3 (27.3%) No: 8 (72.7%)
Coagulation indicators												
PT, s	14.4	17.9	16	15.4	13.8	11.9	28.4	13.7	14.4	13.4	10.5	14.2 (13.48, 15.85)
INR	1.32	1.64	1.47	1.41	1.27	1.09	2.61	1.26	1.32	1.23	0.96	1.30 (1.24, 1.46)
APTT, s	43	60.9	71	77.2	33.9	26.3	58.6	46.1	37.7	30.6	54.3	46.1 (34.85, 60.33)
TT, s	21.3	16.1	19	15.5	20.3	16.7	25.5	13.9	15.7	14.4	14.5	16.1 (14.75, 19.98)
FIB, mg/dL	129	241	330	331	163	354	80	628	304	422	407	330 (182.5, 393.75)
DD, ng/mL	5359	4205	80946	12079	21110	12191	24286	7060	9069	6427	6657	9069 (6484.5, 18880.25)
Platelet, ×10^9^/L	36	8	56	39	42	133	30	113	73	182	44	44 (36.75, 103)
Infection indicators												
PCT, ng/mL	66.8	3.81	28.41	200	8.61	1.28	3.54	4.78	27.07	1.45	0.55	4.78 (1.97, 28.08)
Interleukin-6, pg/mL	540	122	58	5000	3322	134	124	189	561	124	100	134 (122.33, 555.75)
White blood cell, ×10^9^/L	3.7	16.9	9.22	4.81	4.57	28.55	15.17	10.15	20.61	8.12	16.22	10.15 (5.64, 16.73)
Neutrophil, ×10^9^/L	86.1	94.6	88.9	83.5	61.7	93.2	90.6	92.9	95.7	82.1	83.2	88.9 (83.28, 93.13)
Liver and kidney function indicators												
ALT, U/L	80	113	175	59	22	15	14	43	14	17	41	41 (15.5, 74.75)
AST, U/L	76	189	222	155	28	19	67	51	46	98	67	70 (47.25, 140.75)
Total bilirubin, μmol/L	44.1	61.8	168.2	24.8	37.8	4.6	136.1	10.9	36.9	14	22.5	36.9 (16.13, 57.38)
Creatinine, μmol/L	99	56.6	88	223.8	65	612.1	267.5	235.6	260.1	234.1	72.1	223.8 (76.07, 253.98)
Urea nitrogen, mmol/L	8.25	2.69	21.7	10.48	7.78	32.85	19.67	13.08	43	10.5	9.09	10.5 (8.46, 21.19)
Blood gas analysis												
Hemoglobin, g/L	64.7	77.6	90.1	138.2	84.9	68.1	75.1	106.1	93	68	58.5	77.6 (68.03, 92.28)
PH	7.349	7.371	7.344	7.474	7.505	7.359	7.381	7.25	7.41	7.426	7.252	7.37 (7.35, 7.42)
Peripheral blood iCa^2+^, mmol/L	1.31	1.26	1.09	0.94	1.21	0.94	0.76	1.14	1.34	0.98	1.15	1.14 (0.96, 1.25)
Lac, mmol/L	2.8	0.9	1.8	7.4	1.5	5.8	3.9	0.8	2.2	1.2	1	1.8 (1.05, 3.63)
ICU length of stay, days	11	13	9	29	13	14	4	14	29	8	7	12 (8.25, 15.88)
Total length of hospital stay, days	11	22	9	31	45	21	54	14	71	8	7	21 (9.5, 41.5)
Mechanical ventilation time, days	11	2	10	10	8	14	4	6	20	3	0	8 (3.5, 10.8)
CRRT filter life, h	37.5	72	23	65	54	48	39.5	46.5	34	70	72	48 (38, 68.75)
Patient status												
Metabolic acidosis	Yes	No	No	No	No	No	No	No	No	No	No	Yes: 1 (9.1%) No: 10 (90.9%)
Metabolic alkalosis	Yes	No	Yes	No	No	No	No	No	Yes	Yes	Yes	Yes: 5 (45.5%) No: 6 (54.5%)
Hypocalcemia	No	Yes	Yes	Yes	Yes	Yes	Yes	No	No	No	No	Yes: 6 (54.5%) No: 5 (45.5%)
Hyper/hypokalemia	No	No	No	No	No	No	No	No	No	No	No	Yes: 0 (0) No: 11 (100%)
Hyper/hyponatremia	No	No	No	No	No	No	No	No	No	No	No	Yes: 0 (0) No: 11 (100%)
Bleeding	No	No	No	No	No	No	No	No	Yes	No	No	Yes: 1 (9.1%) No: 10 (90.9%)
Restart CRRT within 7 days after off-machine	Yes	Yes	Yes	Yes	No	No	Yes	Yes	Yes	Yes	No	Yes: 8 (72.7%) No: 3 (27.3%)

Measurement data are expressed as median (IQR), enumeration data are expressed as cases (%). T indicates that the patient cannot speak normally due to tracheal intubation or tracheotomy. GCS: glasgow coma scale, RASS: Richmond Agitation-Sedation Scale, BMI: body mass index, AKI: acute kidney injury, ACCI: acute coagulation and inflammation index, SOFA: sequential organ failure assessment, APACHE II: acute physiology and chronic health evaluation II, PT: prothrombin time, INR: international normalized ratio, APTT: activated partial thromboplastin time, TT: thrombin time, FIB: fibrinogen, DD: D-dimer, PCT: procalcitonin, ALT: alanine aminotransferase, AST: aspartate aminotransferase, iCa^2+^: ionized calcium, Lac: lactic acid.

**FIGURE 2:**
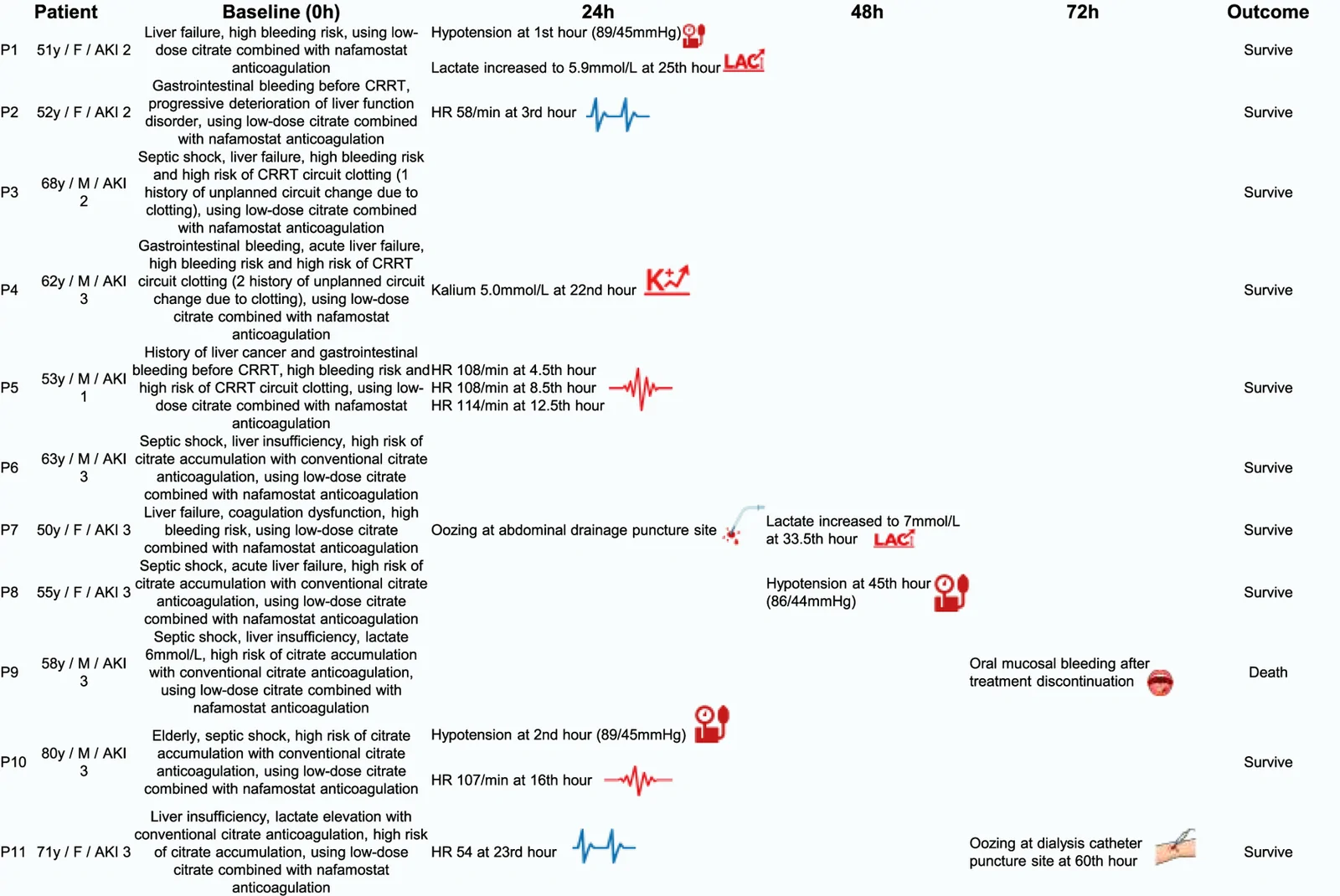
Clinical parameters, monitoring results and outcome information of 11 patients with liver failure receiving low-dose citrate combined with nafamostat mixed anticoagulation CRRT treatment.

Bleeding events were defined as clinically overt bleeding, including but not limited to: bleeding from puncture or catheter sites, oral or nasal mucosal bleeding, gastrointestinal bleeding, hematuria, hemoptysis, or any bleeding requiring transfusion, discontinuation or reduction of anticoagulation, or surgical or interventional hemostasis. Intracranial hemorrhage confirmed by imaging was recorded separately as a major bleeding event. Thrombotic events, including ischemic stroke and filter or circuit thrombosis, were recorded separately as thrombotic complications. Given that coagulation dysfunction is an intrinsic feature of liver failure, baseline APTT, PT-INR, and platelet count were recorded as descriptive baseline characteristics and as safety monitoring parameters during treatment, and were not used to define bleeding events.

Citrate accumulation event was defined as a sustained total-to-ionized calcium (T/iCa) ratio ≥2.5 accompanied by metabolic alkalosis or hypocalcemia. Post-filter ionized calcium was measured to assess the adequacy of regional anticoagulation across the filter (target >0.4 mmol/L), not to define systemic citrate accumulation. Metabolic acidosis event was defined as arterial blood pH < 7.35 and bicarbonate (HCO_3_^−^) <22 mmol/L, excluding the influence of respiratory factors. Metabolic alkalosis event was defined as arterial blood pH > 7.45 and bicarbonate (HCO_3_^−^) >26 mmol/L, excluding the influence of respiratory factors. Hypocalcemia event was defined as peripheral blood ionized calcium (Ca^2+^) <0.90 mmol/L. Hypokalemia: serum potassium <3.5 mmol/L. Hyperkalemia: serum potassium >5.5 mmol/L. Hyponatremia: serum sodium <135 mmol/L. Hypernatremia: serum sodium >145 mmol/L.

Coagulation parameters, including PT, INR, APTT, TT, FIB, D-dimer, and platelet count, were recorded at baseline (before CRRT initiation) and immediately after weaning from CRRT (at off-machine), defined as the first blood draw after circuit disconnection and before any change in anticoagulation or other therapy.

### 2.4 Ethics statement

This study was approved by the Ethics Committee of Zhongnan Hospital of Wuhan University [2026150K]. Because this study is a retrospective study, the Ethics Committee waived the signing of the informed consent form.

### 2.6 Data analysis

SPSS 23.0 statistical software (IBM Corporation, Armonk, New York, USA) was used for data processing in this study. The non-parametric Wilcoxon test was used to test the statistical hypothesis of the distribution difference of quantitative variables in the sample. All statistical tests were two-sided, and P < 0.05 was considered statistically significant. The dynamic changes of inflammatory indicators, platelet count, SOFA score, *etc.*, in patients before and after treatment (T0, T1, T2, T3) with the low-dose regional citrate combined with nafamostat mixed anticoagulation regimen were compared: repeated measures analysis of variance was used for data conforming to normal distribution, and two independent t-tests were performed if there were intra-group differences; Friedman test was used for data not conforming to normal distribution, and Wilcoxon signed-rank test was used for pairwise comparison.

## 3 Results

### 3.1 Demographic data

A total of 11 patients with liver failure undergoing CRRT in CVVHDF mode were included in this study, including 7 males (63.6%) and 4 females (36.4%), with a median age of 58 (52.25, 66.75) years and a median BMI of 22.86 (19.43, 24.86). According to the liver failure phenotypes defined by the 2024 CMA criteria, the study comprised 8 patients with ACLF (72.7%; triggered by sepsis/septic shock in 5, cardiogenic-ischemic injury in 2, and malignant tumor in 1), 1 patient with acute liver failure (9.1%; secondary to gastrointestinal massive bleeding), and 2 patients with subacute liver failure (18.2%). Seven patients (63.6%) had AKI grade 3, median APACHE II score was 23.5 (19.25, 26.88), median SOFA score was 6.67 (4.83, 10), and 7 patients (63.6%) required vasoactive drug support. Baseline information is shown in Table 1, and diagnostic details fulfilled at admission are provided in Supplementary Table S1.

### 3.2 Effect after combined anticoagulation treatment

After treatment with the low-dose regional citrate combined with nafamostat mixed anticoagulation regimen, there were no statistically significant differences in PT, INR, APTT, FIB and D-dimer levels compared with before treatment (P > 0.05); TT was lower than baseline (P = 0.05), and platelet count showed no significant change before and after treatment (P > 0.05), as shown in Table 2; Figure 3A.

**TABLE 2:** Effect of patients after treatment with low-dose regional citrate combined with nafamostat mixed anticoagulation regimen (n = 11).

Parameter	Before treatment (baseline), median (IQR)	After treatment (at off-machine), median (IQR)	P Value
Coagulation indicators			
PT, s	14.2 (13.48, 15.85)	13.5 (12.3, 15.6)	0.656
INR	1.30 (1.24, 1.46)	1.24 (1.08, 1.43)	0.449
APTT, s	46.1 (34.85, 60.33)	47.2 (32, 54)	0.594
TT, s	16.1 (14.75, 19.98)	14.8 (13.2, 16.7)	0.05
FIB, mg/dL	330 (182.5, 393.75)	321 (82, 476)	0.594
DD, ng/mL	9069 (6484.5, 18880.25)	6544 (2411, 25570)	0.328
Platelet, ×10^9^/L	44 (36.75, 103)	46 (32, 129)	0.859
Infection indicators			
PCT, ng/mL	4.78 (1.97, 28.08)	1.92 (0.7, 8.19)	0.005
Interleukin-6, pg/mL	134 (122.33, 555.75)	54 (14.8, 320)	0.131
White blood cell, ×10^9^/L	10.15 (5.64, 16.73)	14.6 (10.95, 25.8)	0.182
Neutrophil, %	88.9 (83.28, 93.13)	90.9 (82.2, 95.3)	1
Liver and kidney function indicators			
ALT, U/L	41 (15.5, 74.75)	23 (19, 42)	0.059
AST, U/L	70 (47.25, 140.75)	53 (40, 98)	0.374
Total bilirubin, μmol/L	36.9 (16.13, 57.38)	43.8 (14, 136.5)	0.241
Creatinine, μmol/L	223.8 (76.07, 253.98)	130.5 (38.3, 175)	0.131
Urea nitrogen, mmol/L	10.5 (8.46, 21.19)	11.05 (5.46, 15.09)	0.657
Disease severity score			
SOFA	6.67 (4.83, 10)	5 (4, 9)	0.463
APACHE II	23.5 (19.25, 26.88)	17 (13, 20)	0.011

Non-normally distributed measurement data are expressed as median (IQR); Wilcoxon signed-rank test was used for comparison before and after treatment; PT: prothrombin time, INR: international normalized ratio, APTT: activated partial thromboplastin time, TT: thrombin time, FIB: fibrinogen, DD: D-dimer, PCT: procalcitonin, IL-6: interleukin-6, ALT: alanine aminotransferase, AST: aspartate aminotransferase, SOFA: sequential organ failure assessment, APACHE II: acute physiology and chronic health evaluation II; P < 0.05 was considered statistically significant. Before treatment (baseline) = before CRRT, initiation; After treatment (at off-machine) = immediately after weaning from CRRT, defined as the first blood draw after circuit disconnection and before any change in anticoagulation or other therapy.

**FIGURE 3:**
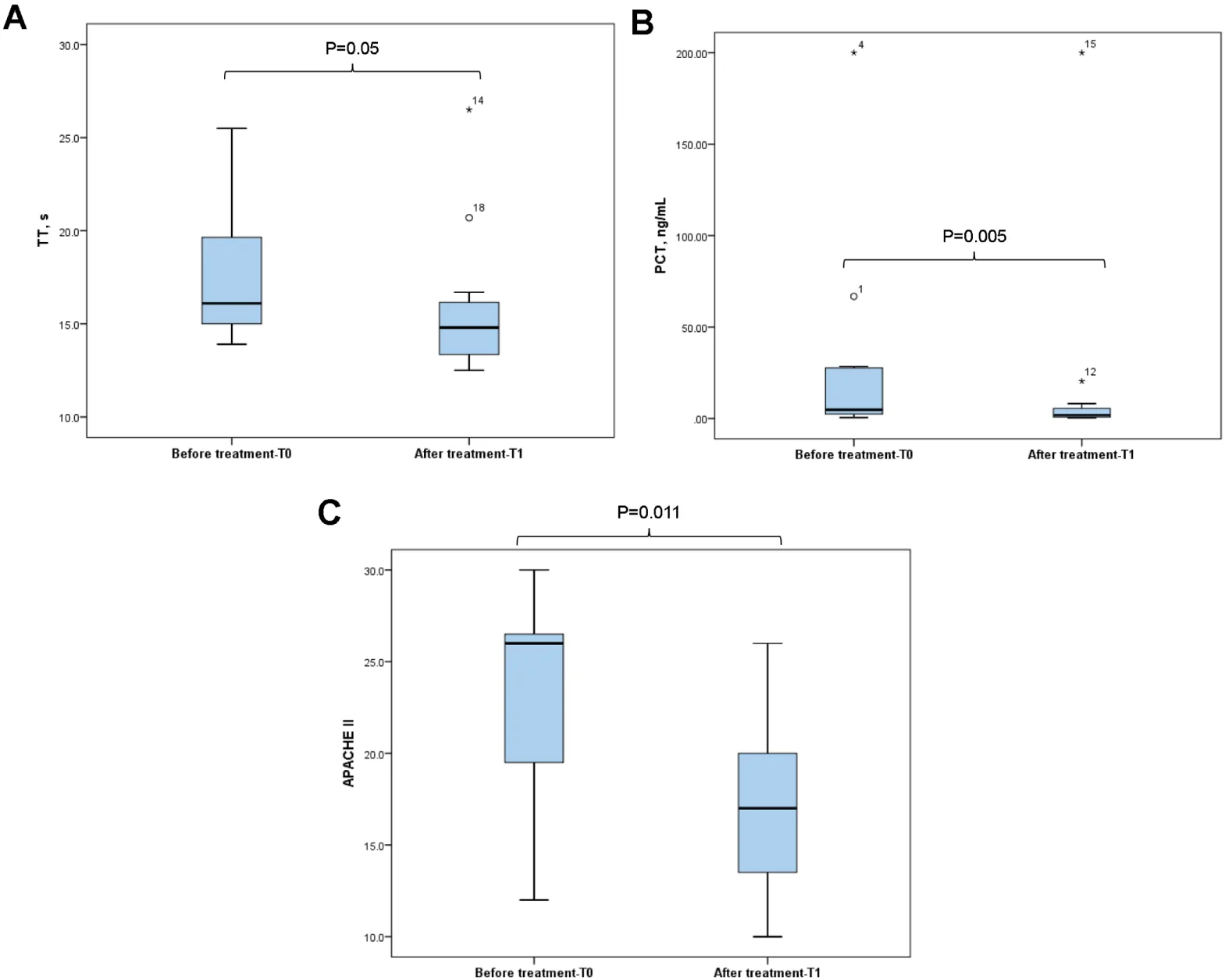
Changes of coagulation indicators, inflammatory biomarkers and organ function severity in patients before and during treatment with low-dose regional citrate combined with nafamostat mixed anticoagulation regimen. **(A)** Comparison of thrombin time (TT) before and after treatment; **(B)** Comparison of procalcitonin (PCT) before and after treatment; **(C)** Comparison of APACHE II score before and after treatment; Wilcoxon signed-rank test was used, *P < 0.05 was considered statistically significant.

After treatment, the PCT level was lower than before treatment (P = 0.005), and there were no significant differences in interleukin-6, white blood cell count and neutrophil ratio compared with baseline (P > 0.05); liver and kidney function-related indicators such as ALT, AST, total bilirubin, creatinine and urea nitrogen showed no significant changes before and after treatment (P > 0.05), as shown in Table 2; Figure 3B.

Compared with before treatment, the APACHE II score of patients was significantly reduced after treatment, with a statistically significant difference (P = 0.011), and the SOFA score decreased but did not reach statistical difference (P = 0.463), as shown in Table 2; Figure 3C.

### 3.3 Patient outcomes

In this study, the median CRRT filter service life of patients was 48 (38, 68.75) hours, up to 72 h; median ICU length of stay was 12 (8.25, 15.88) days, median total length of hospital stay was 21 (9.5, 41.5) days, median mechanical ventilation time was 8 (3.5, 10.83) days. One case (9.1%) had mild oral mucosal bleeding during treatment; no severe massive bleeding, citrate accumulation, severe hypocalcemia or severe acid-base and electrolyte disorders, or other anticoagulation-related serious adverse events occurred. Eight cases (72.7%) restarted CRRT within 7 days after off-machine due to disease needs, and 1 patient died due to severe irreversible disease. The specific information is shown in Table 1; Figure 4. Because no control group was included, these observations are descriptive only and cannot be attributed to the anticoagulation regimen itself**.**

**FIGURE 4:**
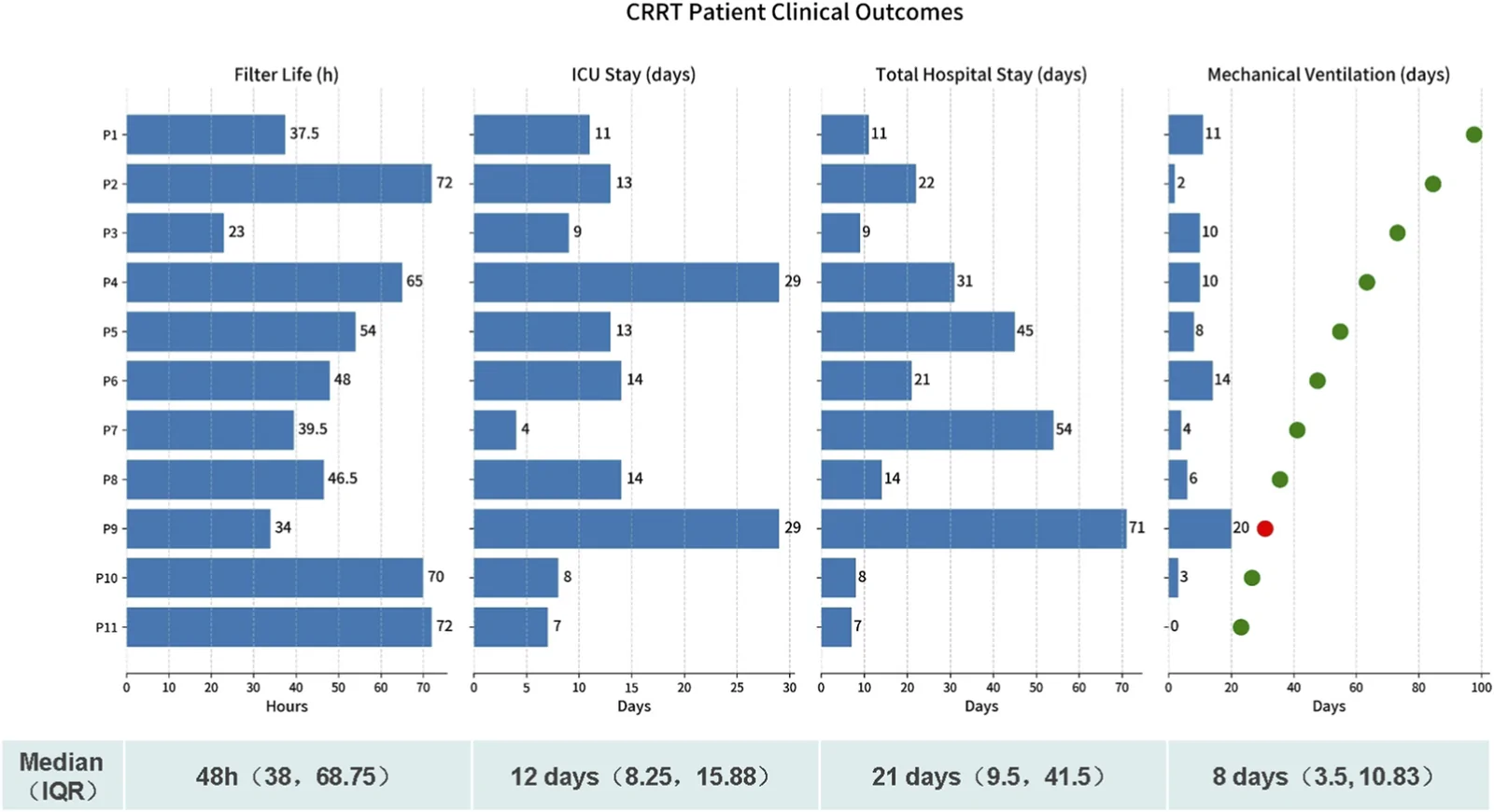
Relevant outcomes of 11 patients (green dot: patient outcome is survival/transfer out (discharge/transfer to other department/transfer to other hospital; red dot: patient outcome is death), data are expressed as median (interquartile range).

## 4 Discussion

This study aimed at patients with liver failure, a high-risk group for CRRT anticoagulation, and describes an exploratory low-dose regional citrate combined with low-dose nafamostat mixed anticoagulation regimen. Through a retrospective case series analysis of 11 cases, we report the operational feasibility of this mode, including its implementation, monitoring, and the adverse events observed. Because of the absence of a control group, no conclusion regarding efficacy or safety relative to other regimens can be drawn.

Patients with liver failure have massive hepatocyte necrosis, severe impairment of liver synthesis, metabolism and detoxification functions, coexistence of coagulation disorders and bleeding risk, and significantly reduced citrate metabolism and clearance capacity, belonging to an extremely high-risk group for CRRT anticoagulation management (Liver Failure and Artificial Liver Group, Chinese Society of Infectious Diseases, Chinese Medical Association, 2025; Expert Group of Chinese Society of Nephrology and Chinese Medical Association, 2022). Previous studies have shown that although conventional-dose RCA can effectively anticoagulate, it is prone to accumulation in patients with liver failure, thereby inducing hypocalcemia, metabolic alkalosis, and even aggravating internal environment disorders (Bai et al., 2024; Ren and Xiang, 2025). While single NM can avoid citrate metabolism problems, it requires a higher dose to ensure filter patency, potentially leading to excessive prolongation of APTT, excessive inhibition of systemic coagulation, and increased bleeding risk (Hu et al., 2024; Zeng et al., 2025). Heparin and low-molecular-weight heparin have a high incidence of bleeding complications in this population due to insufficient synthesis of coagulation factors and thrombocytopenia, and cannot avoid the risk of heparin-induced thrombocytopenia, with poor clinical applicability (Liver Failure and Artificial Liver Group, Chinese Society of Infectious Diseases, Chinese Medical Association, 2025; Expert Group of Chinese Society of Nephrology and Chinese Medical Association, 2022; Ren and Xiang, 2025). In this study, the citrate dose was halved and combined with low-dose NM, with the aim of reducing the liver metabolic burden and accumulation risk through low-dose RCA while reinforcing local filter anticoagulation with NM. Whether this theoretical advantage translates into clinical benefit cannot be determined from the present uncontrolled design.

In terms of anticoagulation efficacy, the median CRRT filter life in this study reached 48 (38, 68.75) hours, up to 72 h. This observation is within the range reported in previous studies of RCA- or NM-based anticoagulation in high-bleeding-risk patients; however, because of the absence of a control group, this observation cannot be attributed to the mixed regimen itself (Kameda et al., 2024; Zhang et al., 2021). Multiple guidelines and clinical studies have suggested that both RCA and NM are recommended for CRRT anticoagulation in high bleeding risk patients, and their combination has been proposed to improve anticoagulation stability without increasing systemic adverse reactions (Expert Group of Chinese Society of Nephrology and Chinese Medical Association, 2022; Hu et al., 2024). The present case series is consistent with the feasibility of this proposal but does not provide comparative evidence.

In terms of safety, there were no obvious abnormalities in systemic coagulation indicators such as PT, INR and APTT in patients after treatment in this study, and only 1 case had mild oral mucosal bleeding; no severe massive bleeding, citrate accumulation, severe hypocalcemia or severe acid-base and electrolyte disorders occurred. Systematic reviews show that the incidence of citrate accumulation in patients with liver failure receiving conventional RCA treatment can reach 5.3% (Qi et al., 2023), and in the study of Choi et al. (2015), the bleeding event rate of NM was 41.9% (13/31). Because our study had no control group, these cross-study figures cannot be used to infer superiority or safety advantage. Through the standardized process of blood calcium stratified calcium supplementation, regular blood gas and APTT monitoring, and precise regulation of drug infusion speed, we maintained anticoagulation intensity at APTT 1.2–1.5 times the baseline value in this cohort. Whether this monitoring strategy reduces adverse events compared with other strategies requires controlled evaluation.

In terms of inflammation and organ function, this study observed that PCT level was decreased and APACHE II score was improved in patients after treatment. It indicates that, on the basis of anticoagulation management, CRRT may clear inflammatory mediators, stabilize the internal environment, and improve organ function. Low-dose citrate did not aggravate the liver metabolic burden in this cohort, and liver and kidney function indicators showed no significant deterioration before and after treatment. These findings suggest that the regimen was tolerated in this small, selected cohort; they do not establish organ-protective effects. Conventional-dose RCA may aggravate internal environment disorders due to accumulation, and high-dose NM may affect the continuity of organ support due to bleeding risk, but whether the present regimen provides comprehensive benefits equivalent or superior to those approaches cannot be determined from this study (Qi et al., 2023; Choi et al., 2015).

The standardized parameters, stratified calcium supplementation, and regular monitoring and regulation process described in this study support the operational feasibility of the mixed anticoagulation regimen. By adjusting calcium supplementation speed according to peripheral blood calcium, and monitoring blood gas analysis and APTT every 2 h within 6 h after initiation, the anticoagulation dose and safety can be adjusted at the bedside, which is convenient for ICU medical staff to implement. This description is intended as an operational reference rather than a validated protocol.

## 5 Limitation

This study has certain limitations: as a single-center, small-sample retrospective study, the level of evidence is limited, no control group was set, and head-to-head comparison with other anticoagulation regimens cannot be performed. The absence of a control group precludes any conclusion regarding efficacy, safety, or superiority over other anticoagulation strategies. No subgroup analysis was conducted according to the cause and severity of liver failure, and the efficacy differences in different populations cannot be clarified. The follow-up time is short, lacking long-term prognosis data. All findings should be interpreted as descriptive and hypothesis-generating. In the future, large-sample, multi-center, prospective randomized controlled studies should be carried out to further verify the universality, long-term safety and impact on patient prognosis of this mixed anticoagulation regimen, and to explore the optimal drug dose combination and monitoring strategy, so as to provide higher-level evidence for CRRT anticoagulation in liver failure.

## 6 Conclusion

This single-center retrospective case series of 11 patients describes the operational feasibility of a low-dose regional citrate combined with nafamostat mixed anticoagulation regimen for CRRT in patients with liver failure. The regimen was implemented at the bedside without severe anticoagulation-related adverse events in this cohort. Because no comparator group receiving conventional RCA, nafamostat alone, systemic heparin, or no anticoagulation was included, whether this regimen prolongs filter life, ensures treatment continuity, or reduces the risks of systemic bleeding, citrate accumulation, hypocalcemia, or acid-base disorders compared with other strategies cannot be determined. In addition, because all patients received concurrent antibiotics, vasopressors, source control, and other ICU supportive care, the observed changes in PCT and APACHE II score cannot be attributed to the anticoagulation regimen. These findings are preliminary and hypothesis-generating and require adequately powered, multicenter, prospective controlled trials. The standardized treatment parameters, drug infusion regimen, stratified calcium supplementation strategy, and blood gas and APTT monitoring process described here may provide a preliminary operational reference for centers considering this strategy, pending validation.

## 7 Implications for clinical practice

Patients with liver failure generally have decreased liver synthetic function, coagulation disorders, and significantly reduced citrate metabolism and clearance capacity, making them a high-risk population for CRRT anticoagulation management. Traditional heparin anticoagulation carries a high risk of bleeding and thrombocytopenia; single conventional-dose citrate is prone to accumulation, hypocalcemia and metabolic alkalosis; single nafamostat is likely to cause excessive inhibition of systemic coagulation and increased bleeding risk. In this case series, we describe an operational approach combining low-dose citrate with nafamostat, together with predefined treatment parameters, drug ratios, a stratified calcium supplementation protocol, and a monitoring and regulation process. This approach was implemented at the bedside in 11 patients with liver failure without severe anticoagulation-related adverse events. However, because this was an uncontrolled retrospective case series, no comparative inference regarding effectiveness or safety can be made. The description provided here may serve as a preliminary operational reference for centers considering this strategy, pending validation in larger controlled studies.

## 8 Contribution of the paper

### 8.1 What is already known

At present, the mainstream anticoagulation methods for CRRT include regional citrate anticoagulation, nafamostat, unfractionated heparin and low-molecular-weight heparin, etc. Each single anticoagulation regimen has obvious shortcomings. Patients with liver failure have impaired liver citrate metabolism, and conventional-dose citrate is prone to accumulation inducing hypocalcemia and acid-base imbalance; single nafamostat has difficulty in controlling the systemic anticoagulation dose, easily causing excessive prolongation of APTT and increasing bleeding risk; heparin has a high incidence of bleeding complications in people with coagulation disorders due to liver failure. Existing studies mostly focus on the comparison of single anticoagulation regimens, and there is still a lack of studies on standardized parameters, monitoring and management system, and clinical efficacy and safety of low-dose citrate combined with nafamostat mixed anticoagulation specifically for the simple liver failure population.

### 8.2 What this paper adds

- Describes an exploratory low-dose regional citrate combined with nafamostat mixed anticoagulation mode, designed to accommodate the pathophysiological characteristics of decreased citrate metabolism and fragile coagulation in patients with liver failure.
- Reports a set of CVVHDF treatment parameters, drug infusion doses, a stratified calcium supplementation protocol, and blood gas and APTT monitoring frequency and regulation targets that were implemented at the bedside in this case series.
- Based on 11 retrospective cases, shows that this regimen was operationally feasible, with no severe anticoagulation-related adverse events observed in this cohort; however, because of the uncontrolled design, these findings are descriptive and hypothesis-generating and do not establish efficacy, safety, or superiority over other anticoagulation strategies.
- Provides a preliminary operational reference and a basis for designing subsequent large-sample controlled studies, and offers new ideas for CRRT anticoagulation strategies in similar critically ill patients, pending validation in adequately powered, multicenter, prospective controlled trials.

## Data Availability

The original contributions presented in the study are included in the article/Supplementary Material, further inquiries can be directed to the corresponding authors.
