# Supplementary material for: Successful application of low-dose regional citrate combined with nafamostat mixed anticoagulation in CRRT for patients with liver failure: a retrospective case series study of 11 patients
Source: Front Pharmacol. 2026 Oct 21;17:1896125. doi: 10.3389/fphar.2026.1896125 (PMC13730937; doi:10.3389/fphar.2026.1896125)
Supplement: Supplementary file 1 [file Table1.docx]

**Supplementary Table S1**

**Liver failure phenotype, underlying chronic liver disease, timing of diagnosis, and key diagnostic criteria fulfilled at admission for each patient.**

| **Patient** | **Phenotype** | **Etiology / trigger** | **Underlying chronic liver disease** | **Timing of diagnosis** | **HE grade** | **INR / PTA** | **TBil at diagnosis (μmol/L)** | **TBil trajectory** | **Other criteria fulfilled** |
| --- | --- | --- | --- | --- | --- | --- | --- | --- | --- |
| P1 | ACLF | Sepsis | Yes | At admission | II | INR 1.8 | 236 | Declined after ICU treatment | Ascites, AKI |
| P2 | ACLF | Gastrointestinal massive bleeding | Yes | At admission | II | INR 1.7 | 215 | Declined after ICU treatment | Ascites |
| P3 | Acute liver failure | Septic shock | No | At admission | III | INR 2.1 | 320 | Declined | HE grade III |
| P4 | ACLF | Septic shock | Yes | At admission | II | INR 1.6 | 228 | Declined | AKI, shock |
| P5 | ACLF | Malignant liver tumor | Yes | At admission | II | INR 1.6 | 212 | Declined | Malignant liver tumor, AKI |
| P6 | ACLF | Septic shock | Yes | At admission | II | INR 1.9 | 265 | Declined | AKI, shock |
| P7 | Subacute liver failure | Hepatitis/chronic liver disease \| | Yes | At admission | II | PTA 38% | 198 | Declined | Ascites |
| P8 | ACLF | Septic shock | Yes | At admission | II | INR 1.8 | 242 | Declined | AKI |
| P9 | ACLF | Peritoneal cancer | Yes | At admission | II | INR 1.6 | 209 | Declined | Peritoneal cancer, ascites |
| P10 | ACLF | Cardiogenic shock | Yes | At admission | II | INR 1.7 | 221 | Declined | Shock, AKI |
| P11 | ACLF | Cardiogenic shock | Yes | At admission | II | INR 1.6 | 208 | Declined | AKI |

**Abbreviations:** ACLF, acute-on-chronic liver failure; HE, hepatic encephalopathy; INR, international normalized ratio; PTA, prothrombin activity; TBil, total bilirubin; AKI, acute kidney injury; ICU, intensive care unit.
